# Hospital and Institutional News

**Published:** 1921-01-08

**Authors:** 


### HOSPITAL AND INSTITUTIONAL NEWS.

### NEW YEAR HONOURS.

The feature of the New Year Honours which will give as much pleasure as any to hospital people is the award of the Grand Cross of the Victorian Order to the Duke of York. His Royal Highness has much identified himself lately in hospital affairs, and his sensible and practical speeches at hospital meetings have been keenly appreciated. The peerage bestowed upon Sir Horace Brooks Marshall, Lord Mayor of London in Armistice year, is another well-deserved honour which will give much pleasure to all who know of Sir Horace's labours over many years for a multitude of good works. Not merely from the journalist point of view must we welcome the inclusion in the list of Dr. Samuel Squire Sprigge, the editor of the *Lancet*, and Dr. Dawson Williams, editor of the *British Medical Journal*. Medical men and hospital workers in the honours list are not numerous, but we note that Sir Andrew Beattie, D.L., ex-High Sheriff of Dublin City, who is a member of the Council of the College of Nursing, has been made a Privy Councillor; that Mr. Donald Macmaster, K.C., M.P., who, as Chairman of Standing Committee E of the House of Commons on Nurse Registration, devoted much time in the spring of 1919 to the cause of the nursing profession, has been made a Baronet; and that Mr. Charles Ryall, C.B.E., F.R.C.S., senior surgeon of the Cancer Hospital and the Bolingbroke Hospital, becomes a Knight Bachelor. Mr. Pietro Michelli, C.M.G., the secretary of the London School of Tropical Medicine and the Seamen's Hospital Society, Greenwich, also receives the same honour. Sir Pietro is now one of our oldest hospital workers, having been appointed secretary to the Seamen's Hospital in 1887, while he was secretary of St. Mary's Hospital for six years previously; we gladly join in the volume of congratulations that must be pouring in from his host of friends and well-wishers. A Knighthood has also been conferred on Dr. E. Coey Bigger, Chairman of the Public Health Council and Medical Member of the Local Government Board, Ireland, who played an important part in connection with the passing of the Midwives Act for Ireland.

### THE NATIONAL RELIEF FUND.

We understand from the Secretary of the National Relief Fund that particulars of the recent grants to hospitals to reduce war deficits are not at present to be issued. Decisions have been communicated to all hospitals concerned, and details of the grants (including the Scottish and Irish distributions, which have not yet been completed) will be published in the Committee's report in due course. As the whole of the share of the grant applicable to England and Wales has now been allocated, the Committee will be unable to undertake to review the decision arrived at in any particular case. The Great Northern Central Hospital has received £9,250, and the National Hospital for the Paralysed and Epileptic £4,200.

### COLONEL DEAN'S SUCCESSOR AT BELFAST.

Colonel John Vincent Forrest, C.B., C.M.G., has been appointed superintendent of the Royal Victoria Hospital, Belfast, in succession to Colonel Dean, who lately retired in consequence of ill-health. The new superintendent, who is assistant-director of medical services in the Dublin district, and is still on the active service list, is about to retire from the Army. For the past six years he has been administrator of military hospitals, and during the war was D.D.M.S. of the Hospital Base at Boulogne. Under him were a score of hospitals and convalescent camps, and it was for his services in connection with the education of wounded that he received the C.M.G. In 1917 Colonel Forrest was transferred to Italy in charge of the medical arrangements on the lines of communication from Italy to the East. Still under fifty years of age, Colonel Forrest graduated at Edinburgh University, and is well qualified to continue Colonel Dean's work in the organisation of voluntary contributions to the Royal Victoria Hospital, which is perhaps the most famous institution of its class in Ireland.

### ST. MARY'S HOSPITAL, PLAISTOW, A SORRY DECISION.

We regret to see that the committee of St. Mary's Hospital for Women and Children, Plaistow, has decided to reduce the number of wards. The excuse for this decision is, of course, financial need, though the sum is relatively small. Probably £5,000 would put it on its feet again. The effect will be to reduce the number of beds by twenty—from forty-three to half their present number. Plaistow is a district which could well maintain the hospital, were it only properly organised. What efforts have been made in this direction? Have all those works and shops unaffiliated to the Hospital Saturday Fund been approached for regular contributions? If not, why not?

### THE TURKEYS AT THE LONDON.

At first, for financial reasons, it was decided by the committee of the London Hospital with great regret that turkeys were to be absent from the patients' dinner on Christmas Day, although, by some reasoning which is not apparent, the nurses were to have them. We rather think the nurses would have preferred the arrangement turned the other way round; anyhow, they brought to bear a policy of pinpricks on Lord Knutsford, which eventually brought about a universal turkey dinner. Seemingly the point which was most telling was that as some of the wards received birds as presents it was awkward to turn other wards into birdless groves at the festive season. Moreover, as Lord Knutsford says: "What house-physician or house-surgeon will dress himself up in cap and apron to carve beef or mutton or cod at the 'London' on Christmas Day?" What a delightful Knutsfordian touch is the "or cod," which is a distinct contribution to the gaiety of hospitals and welcome in these days! Most hospitals must have gone through a turkey crisis, but mercifully most received special gifts of money towards the cost of Christmas cheer, and were in a position to order the birds without many twinges of conscience.

### THE DEPENDANTS OF DOCTORS.

The ladies responsible for the management of the Royal Medical Benevolent Fund Guild are making a serious effort to raise more money for grappling with the numerous applications they receive for help in the matter of the education of the children of those members of our profession who have died or lost their health before they have been able to make adequate provision for their dependants. We recently announced the result of the Matinée held a month ago at His Majesty's Theatre, and now we have to report that a very successful dance was held on December 23 at 64 Wimpole Street. The owner of the house generously bore all the expenses, including the band and refreshments, so that the small committee of ladies had nothing to do but sell the tickets. These were limited to 105 at a guinea each, and they were all sold very quickly. We mention these details as it is obvious that here we have an example of combining charity with pleasure which might well be followed by other doctors who are the fortunate possessors of suitable houses either in London or in the provincial towns, and it is hoped that many will make a similar effort and thus give their patients an opportunity of materially assisting the less fortunate members of our profession. Anyone who is willing to lend his house for the purpose of a dance is invited to communicate with the Secretary of the R.M.B.F. Guild, 19 Portland Place, W. 1, giving some indication of how much of the expenses he is prepared to contribute and of the number of couples his rooms will accommodate, with the price at which he thinks the tickets would be likely to sell in his neighbourhood.

### A HOSPITAL TRUSTEE RESIGNS.

Sir David Salomons has resigned his trusteeship of the Tunbridge Wells General Hospital, and proposes to withdraw his support until the Committee of Management shall have adopted adequate means to meet the serious annual deficit. This appears to be the action of a friend who thinks that the time has come to give a jog to hesitation. The Committee appears guilty of acts of omission, nothing more, but nothing less. In lately declining to adopt a system of contributions towards maintenance the Committee has our sympathy, whatever views of its wisdom may be held, but this decision was accompanied by a proposal so very modest as to merit no record here. It was this latter suggestion that Sir David seems to have thought trifling. In our view, the solution would be, not contributions from patients, but rather contributions from employers and employed. The latter, being less easy to collect, are less popular, but infinitely more promising, besides being in the voluntary tradition. They only require a little brains and determination, in proof of which many examples can be quoted from districts as different as is Chichester from Leeds. We invite Sir David Salomons to crown his act of decision by another, and to study the system of Chichester and Leeds before relying wholly upon patients' contributions. It should be a race between him and the Committee to see who can master the situation first.

### TONSILS AND ADENOIDS IN THE O.P. THEATRE

For very many years it has been customary to regard all ordinary uncomplicated cases of removal of tonsils and adenoids in childhood as suitable for the out-patient theatre, and to allow the parents to remove the children after an interval for recovery from the anæsthetic. The practice is admitted not to be so desirable as in-patient treatment would be; but in most hospitals the question of beds dominates the situation, for at least twenty times as many sufferers can be relieved by O.P. operation as by admission to the wards. In a contribution to the *British Medical Journal* Mr. E. Watson-Williams draws attention to some of the drawbacks to the performance of tonsillectomy in the O.P. theatre. Analysing a series of 339 patients treated at the Bristol Royal Infirmary, he concludes that the morbidity of in-patient cases is about 1 per cent., that of O.P. cases over 4 per cent. (the mortality is, of course, much lower for each class). He concludes: "Although the point has been recognised for years, I should like to ask whether sufficient weight is given to it, and what, if any, variation in present practice is desirable." The problem is difficult, but the answer he will most likely get is that hospitals have to study the greatest good of the greatest number.

### A GOOD WEEK OF BEQUESTS.

One should be careful to observe, when a reversionary interest falls in, that the legatees get nothing in the shape of a surprise, and that they have already probably discounted their interest. All the same, the large sum of £462,000 divided amongst seven institutions on the death of Mrs. Gorringe comes at a time of financial stringency, and will be very welcome. The hospitals concerned are St. George's and Westminster, which will receive, roughly, only £25,000 each, as the securities of the estate have depreciated in value by 50 per cent. Another interesting will is that of Mr. Walter Hutton, author of several works on engineering, who recently died at Eastbourne at the age of eighty-one. At St. Thomas's Hospital, where Mr. Hutton's late son received his medical education and was house physician and surgeon in 1887, a bed is endowed In Memoriam, and beds in memory of the testator's daughters and other relations are to be endowed or named at the Victoria Hospital for Children, the Great Ormond Street Children's Hospital, the Royal Waterloo Hospital, the Middlesex Hospital, the Great Northern Central Hospital, the Evelina Hospital, and the Lock Hospital.

### THE DECLINE OF DRUNKENNESS.

According to Dr. Hope, the medical officer of health for Liverpool, the welcome decrease previously noted in the number of deaths due to, accelerated by, excessive drinking has been maintained. Moreover, he points out that the beneficial results of the restricted manufacture and sale of intoxicating drink are not to be measured by a reduced mortality only, for there is also a great decrease in the number of cases of *delirium tremens* coming under treatment in public institutions. The decrease in the mortality from excessive drinking, which is especially noticeable in regard to females, has been followed by a decrease in the number of infants dying from suffocation caused through overlying by their parents. Drink deaths, which in 1914 were 125, were reduced to 19 in 1919, whilst suffocated infants were 25 only, compared with 76 in 1914.

### AN OVERDUE REFORM IN ASYLUMS.

The medical consultant to the Western Command, Lieut.-Colonel Ernest White, C.B.E., M.B., who was from 1887 to 1905 medical superintendent of the City of London Asylum, has made an interesting report to the Director-General of the Army Medical Service upon the Whitchurch Mental Hospital during its occupation by the military. The institution, known then as the Welsh Metropolitan War Hospital (Mental Division), was, he says, *facile princeps* of all the institutions of its class. The structure is "new," the equipment "scientifically complete," and the wards are small and accommodate forty instead of the usual 120. In fact, the total number of patients was only 450, "a workable number," as Dr. White expresses it. A feature of each ward is the verandah for open-air treatment. Dr. E. Goodall was in command. The wards, Dr. White reports, were controlled by female nurses with the help of one or two orderlies. The nursing was "sympathetic," and, in consequence, "everything worked harmoniously." It can be understood why Dr. White holds that this system should be generally adopted for the insane, and certainly the small ward is an overdue reform in asylums.

### TWO YEARS' FRUIT AT BLACKBURN.

The delegates from all representative trades in Blackburn and East Lancashire were invited to attend the annual meeting of the East Lancashire Workpeople's Hospital Fund last week. The fund, of which Mr. Herbert Holden is secretary, has now presented its second report on the work started in September 1918. In the first complete year over £4,000 was contributed by the workpeople to the Royal Infirmary, Blackburn. In the second the total has risen to about £10,500, a remarkable increase, which shows the healthy condition of the Fund. The Committee of the Fund has sent deputations to meetings of trades unions, and local workpeople's funds have been encouraged by its means. Wherever, we learn, an opportunity has been given to explain the scheme, "it has invariably been adopted." But the complete success of the scheme depends upon the active co-operation of the employers, and those who at present are content with occasional collections for the hospital must help their staffs to arrange regular contributions, and themselves to subscribe toward them. The chairman, Mr. Lawrence Cotton, and Mr. Herbert Holden may well be proud of their two-years-old achievement, and we hope that their public spirit will infect others, especially employers. At the present moment example is more valuable than ever, and those who set it deserve unstinted praise.

### DR. BARNARDO'S MATCHES.

So far as we remember, Dr. Barnardo's Homes are the only institution to provide the public with a pleasant excuse for dropping something into a collecting-box. You are at a restaurant, and after the walnuts and the wine you want a match. You call the waiter, who brings a collecting-box attached to which is a tray containing, in the French fashion, a set of flat matches in cardboard wrappers. It would be indecent to requite this service with less than a penny, and we suppose that in practice such loose pence as happen to be in your pocket are dropped into the box. The writing on the wrapper or packet invites you, when you have used all the matches, to send a contribution to the Homes. Whether you do or do not you are grateful for the institution's thought of your convenience, and amused at its enterprise and foresight. It would be interesting to know what is the revenue derived from the sale of these matches, and how many donors they bring. The plan has been in existence for some time, but we think that it has never been copied. Dr. Barnardo's Homes have indeed a prescriptive right to the idea, but the idea might be copied in regard to other small commodities besides matches. Perhaps our readers can suggest a better application of the principle.

### AN UPHILL FIGHT AT PORTSMOUTH.

It seems to be rather an uphill fight at Portsmouth to bring all local industries into the weekly contribution scheme. Apparently, for one reason or another the men in the dockyard, while not hanging back, have been more slow to organise themselves, though the scheme is favoured by them. It is good to learn that £5,000 a year is expected by Mr. B. Wagstaff, the secretary, from this source, and that the number of contributions does not seriously decline. The scheme in fact hardly was set on its feet till last July, and the hospital's income for November was £1,225, compared with £870 a year ago. The contributors should be encouraged to observe that the waiting-list is low, only fifteen in December. Consequently, the hospital does its work, and it is for the contributors to enable it to continue.

### A CONTRIBUTION SCHEME AT BRISTOL.

The finance of the Bristol Royal Infirmary has always been interesting, because the late chairman, Sir George White, when the new buildings were proposed favoured the realisation of all investments, and relied upon a vast body of annual subscriptions to maintain the new institution. It was a bold policy, because annual subscriptions are always hard to secure in anything like sufficient quantity. Sir George, however, did collect much support from this source, but now, with a deficit of £60,000, a sum still rising fast, workers' contributions are a necessity. Already the staff of Messrs. Wills, tobacconists, raise £2,000, which it divides among medical charities of the city. The men in the Albert Dockyard subscribe in the same way £1,500 a year, and Mr. Grimston, of the Dockers' Union, whose members number 40,000, hopes to induce them to join the scheme. If each member gave 2d. a week £8,000 would be raised by them alone. At a recent meeting of delegates from 100 firms in Bristol, at which Mr. Grimston spoke, it was resolved to invite all the workers to subscribe on an organised plan, and in return to secure the representation which is much prized in Bristol. We hope that the scheme will be carried through. The movement is spreading. Why should Bristol leave itself in the lurch?

### TAUNTON HOSPITAL'S DECISION.

The Committee of the Taunton and Somerset Hospital has adopted the suggestion of the Chairman, Mr. R. Vernon, to impose a minimum charge of two shillings a day toward the cost of maintenance of all patients able to pay. This is not a payment, but a contribution toward the cost, which of course far exceeds this sum. It is to be noticed that the proceeds of the hospital-week scheme, started three years ago, have declined. It raised at first £1,300, then £1,000, then only £650. Consequently, organisation is at fault, and we think this is evident in the Committee's decision to enforce the rule for recommendations. We have never favoured these, and hope more from regular contributions from the well than from any other source.

### A CONFERENCE ON CO-ORDINATION.

Recently the Willesden Urban District Council authorised a conference comprising six councillors, six members of the local Board of Guardians, three representatives of the Willesden Cottage Hospital, and three representatives of the Willesden branch of the British Medical Association to consider the future provision of medical and allied services. This conference has now been held. The medical officer of health pointed out that with so many independent bodies and authorities carrying out medical work in the same area it is impossible to prevent overlapping, and it was to the elimination of this that the conference chiefly confined its discussions. The representatives of the British Medical Association intimated that the branch accepts the broad principles of and has decided to follow on the lines of the Interim Report of the Consultative Council on Medical and Allied Services. The conference passed a resolution expressing the opinion that it is desirable that Willesden, with its population of 171,000, should be a distinct unit for all its health services, and a sub-committee was appointed to secure this object.

### CHRISTMAS GIFTS PER FIRE ENGINE.

Perhaps for novelty in the distribution of Christmas gifts to the patients in their isolation hospitals it would be difficult to beat the method adopted by the local District Council at Walthamstow, where on Christmas morning the fire engine was requisitioned for the purpose, and carrying Santa Claus in the person of the Chairman of the Health Committee, was driven first to the Sanatorium at Chingford and then to "Brookfield" Hospital at Hale End, where in each case seasonable and useful gifts were left for distribution to the patients who, thanks to the untiring energies of the staffs, spent a very enjoyable and happy time.

### FIVE POUNDS PER BED PER WEEK.

It was stated at a meeting of the Croydon Council that the estimate of the expenditure of the Croydon Borough Infectious Hospital would be £25,000 for the coming year, or on the basis of 100 occupied beds per day, each patient would cost £250 in the year. The accounts showed that the cost of provisions per week for each patient is 30s., which, it was submitted by Alderman Trumble, who brought the matter forward, notwithstanding the admitted increased cost of living, seemed much too high. An investigation of the expenditure was asked for.

### THE "STIGMA" OF THE DISPENSARY.

Dr. Bell Furguson, the tuberculosis officer of York, reporting on the work of the dispensary, says the majority of patients attend willingly, but a few seem to fear a stigma will attach to them if they have ever been examined at a "tuberculosis dispensary." It is, he says, worth considering whether such an institution should not be labelled rather "clinic for diseases of the chest," and work in close connection with the out-patient department of a general hospital. He also mentions that at times they have failed to induce patients to enter an institution when a bed has been offered, and suggests that compulsory powers of isolation would be desirable to prevent the spread of the disease to young children especially, where the patient cannot or will not take the necessary precautions. On this latter point, by the way, Dr. Wright Mason, the medical officer of health for Hull, states that if advanced cases are in crowded homes, or homes where there are young children, they ought to be removed to prevent them infecting others. It is most desirable that a separate hut should be provided in which such advanced and infectious cases can be accommodated.

### THIS WEEK'S DRUG MARKET.

The position is much the same as last week except that there is just a little more buying; this, however, is on a small scale only, and at present there are no indications of an impending revival in the drug trade or that the easier movement in prices is likely to receive an immediate check. It is considered that some of the synthetic drugs have reached a price-level below which they are not likely to go much further, but if this view were widely held buying on a larger scale would be commencing, and at the moment it is not. The bogey of German competition is making some operators timid, and it certainly seems likely that competition from this quarter will increase. In crude drugs there is very little doing and stocks are increasing.

